# Assessment of Nutritional Status, Dietary Strategies and Selected Biochemical Indicators in Gastrointestinal Cancer Patients: Clinical Implications for Tertiary Prevention

**DOI:** 10.3390/biomedicines14071518

**Published:** 2026-07-06

**Authors:** Kamil Michał Mąkosza, Janusz Wierzgoń, Małgorzata Muc-Wierzgoń, Sylwia Dzięgielewska-Gęsiak

**Affiliations:** 1 Doctoral School, Medical University of Silesia in Katowice, 40-055 Katowice, Poland; 2 Department of Internal Diseases Propaedeutics and Emergency Medicine, Faculty of Public Health in Bytom, Medical University of Silesia in Katowice, 41-902 Bytom, Poland; mwierzgon@sum.edu.pl (M.M.-W.); sgesiak@sum.edu.pl (S.D.-G.); 3 First Department of Surgical Oncology, National Institute of Oncology, 44-102 Gliwice, Poland; janusz.wierzgon@gliwice.nio.gov.pl

**Keywords:** gastrointestinal cancers, nutritional status, dietary strategies, hidden malnutrition, inflammatory biomarkers, nutritional profiles, tertiary prevention

## Abstract

**Background**: Nutritional deterioration and systemic inflammation frequently accompany gastrointestinal cancers and may negatively affect treatment tolerance, quality of life, and clinical outcomes. This study aimed to evaluate nutritional status, dietary behaviors, inflammatory biomarkers, and multidimensional nutritional–inflammatory profiles in patients with gastrointestinal cancers within the context of tertiary prevention. **Methods**: A cross-sectional study was conducted among 150 patients with gastrointestinal cancers. Nutritional status was assessed using the Mini Nutritional Assessment (MNA), while dietary behaviors were evaluated using an original questionnaire. Biochemical markers, including albumin, hemoglobin, C-reactive protein (CRP), fibrinogen, and neutrophil-to-lymphocyte ratio (NLR), were evaluated in participants with available laboratory data. Exploratory hierarchical clustering analysis was performed to identify multidimensional nutritional–inflammatory profiles. **Results**: According to the MNA classification, 79.3% of participants were at risk of malnutrition and 2.0% were malnourished despite predominantly normal or excessive body weight. Nutritional risk was identified in 91.4% of patients with normal BMI and in 79.5% of overweight patients. Only 32.0% of patients reported receiving dietary counseling during treatment, while oral nutritional supplements and therapeutic diets were used by 40.7% and 41.3% of participants, respectively. Biochemical analyses revealed elevated inflammatory markers accompanied by reduced albumin concentration and anemia-related abnormalities. Exploratory clustering analysis suggested three distinct nutritional–inflammatory profiles (Stable/Supported, Hidden Malnutrition, and Inflammatory Deterioration), highlighting metabolic heterogeneity within the study population. **Conclusions**: Patients with gastrointestinal cancers frequently present nutritional risk accompanied by inflammatory activation despite preserved or excessive body weight. A multidimensional assessment integrating nutritional screening, dietary evaluation, inflammatory biomarkers, and exploratory profile-based clustering may improve understanding of nutritional heterogeneity in gastrointestinal cancer patients and may support future research on individualized nutritional assessment and supportive care.

## 1. Introduction

Gastrointestinal cancers remain among the leading causes of cancer-related morbidity and mortality worldwide and constitute a major clinical and public health challenge [1,2,3]. Gastrointestinal malignancies, particularly colorectal, gastric, pancreatic, esophageal, and liver cancers, are associated with a high symptom burden, metabolic alterations, systemic inflammation, and progressive deterioration of nutritional status during the course of the disease and anticancer treatment [4,5,6]. The prevalence of malnutrition in patients with gastrointestinal malignancies ranges from 30% to more than 80%, depending on tumor location, disease stage, and treatment modality [4,5,6,7,8].

Cancer-related malnutrition is a multifactorial condition resulting from reduced dietary intake, systemic inflammation, metabolic dysregulation, and treatment-related adverse effects [4,5]. In patients with gastrointestinal cancers, impaired nutritional status may be further aggravated by disturbed digestion and absorption, anorexia, nausea, vomiting, diarrhea, constipation, dysphagia, xerostomia, taste and smell disturbances, and early satiety [9,10,11]. These complications frequently lead to insufficient energy and protein intake, progressive body weight loss, sarcopenia, and cancer cachexia [12,13,14]. Consequently, nutritional deterioration may adversely affect treatment tolerance, postoperative recovery, hospitalization duration, functional status, and overall prognosis [6,12,15]. Therefore, early nutritional assessment and continuous monitoring may play an important role in tertiary prevention aimed at reducing treatment-related complications and improving clinical outcomes in cancer patients.

**Figure 1:**
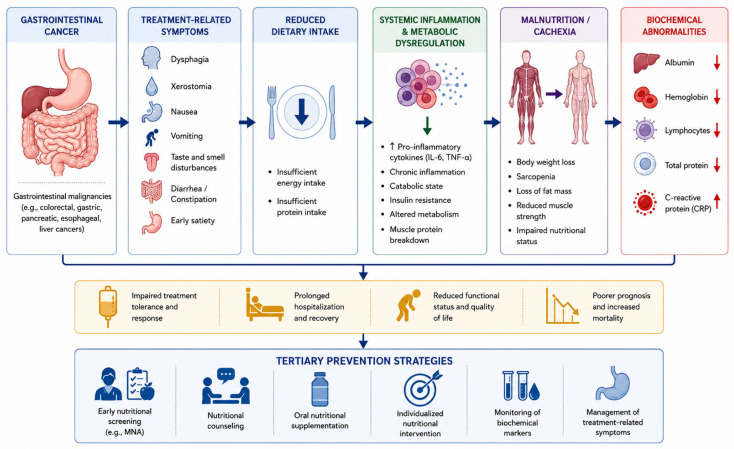
Interrelationships among gastrointestinal cancer, treatment-related symptoms, systemic inflammation, malnutrition, biochemical abnormalities, and tertiary prevention strategies.

Systemic inflammation plays a central role in the pathogenesis of cancer-associated malnutrition and cachexia. Chronic activation of inflammatory pathways contributes to catabolic processes, skeletal muscle wasting, impaired protein synthesis, appetite suppression, and metabolic imbalance [14,16]. Pro-inflammatory cytokines, including interleukin-6 (IL-6) and tumor necrosis factor alpha (TNF-α), contribute to accelerated protein degradation, progressive skeletal muscle wasting, and nutritional decline in cancer patients [16]. Moreover, inflammation-related metabolic disturbances may significantly influence biochemical parameters commonly used in nutritional assessment [17]. The interrelationships among gastrointestinal cancer, treatment-related symptoms, systemic inflammation, nutritional deterioration, and biochemical abnormalities are presented in Figure 1.

Current recommendations of the European Society for Clinical Nutrition and Metabolism (ESPEN) emphasize the importance of early nutritional screening and individualized nutritional intervention in cancer patients, particularly in those with gastrointestinal malignancies who are at high risk of malnutrition [4,5]. However, assessment of nutritional status should not rely solely on anthropometric measurements such as body mass index (BMI), as BMI alone may inadequately reflect nutritional deterioration in patients with sarcopenia, systemic inflammation, or cancer cachexia [12,18]. Therefore, multidimensional evaluation combining clinical, anthropometric, dietary, and biochemical parameters is currently recommended [5,18,19].

Appropriate nutritional management in cancer patients may include nutritional counseling, individualized dietary modifications, oral nutritional supplements, and nutritional support strategies adjusted to treatment-related symptoms and metabolic disturbances [5,18,20]. Previous studies indicate that inadequate nutritional education and limited access to professional dietary support may contribute to insufficient nutritional intake and worsening nutritional status during anticancer therapy [18,21,22].

Biochemical indicators such as serum albumin, total protein concentration, hemoglobin level, lymphocyte count, white blood cell count, and C-reactive protein (CRP) are commonly used in the assessment of nutritional and inflammatory status in cancer patients [5,17,20]. These biomarkers may reflect both nutritional deterioration and the intensity of the systemic inflammatory response associated with cancer progression. Previous studies suggest that abnormalities in biochemical and inflammation-related parameters are associated with poorer clinical outcomes, impaired treatment tolerance, and increased mortality among patients with gastrointestinal cancers [6,15,17].

Despite growing interest in nutritional care in oncology, data integrating clinical nutritional assessment, dietary assessment, and biochemical indicators in patients with gastrointestinal cancers remain limited [18,19,20,21,22]. Therefore, the present study aimed to evaluate nutritional status, dietary behaviors, nutritional support strategies, inflammatory biomarkers, and nutritional–inflammatory profiles in patients with gastrointestinal cancers within the context of tertiary prevention and supportive oncology care.

## 2. Materials and Methods

### 2.1. Study Design and Participants

This cross-sectional observational study included 150 patients (*n* = 150) diagnosed with gastrointestinal cancers. Data collection was conducted between 2024 and 2026 in two clinical centers located in the Silesia region, Poland.

The study population consisted of adult patients with histopathologically confirmed gastrointestinal malignancies, including colorectal, gastric, pancreatic, esophageal, and liver cancers. Eligible participants were required to be aged ≥18 years, receive oral nutrition, remain in a stable clinical condition enabling participation in the study, and provide informed consent prior to enrollment. Additionally, patients with diagnosed neurological or psychiatric disorders potentially affecting rational understanding and questionnaire completion were excluded from participation.

The exclusion criteria included inability to provide informed consent, severe cognitive impairment preventing questionnaire completion, enteral or parenteral nutrition, and withdrawal from the study at any stage.

The study was conducted in accordance with the principles of the Declaration of Helsinki and applicable regulations concerning personal data protection and patient rights. According to the official statement issued by the Bioethics Committee of the Medical University of Silesia in Katowice, the questionnaire-based study did not constitute a medical experiment and therefore did not require formal approval of the Bioethics Committee (decision no. BNW/NWN/0052/KB/145/I/24).

All participants provided informed consent before participation in the study.

**Figure 2:**
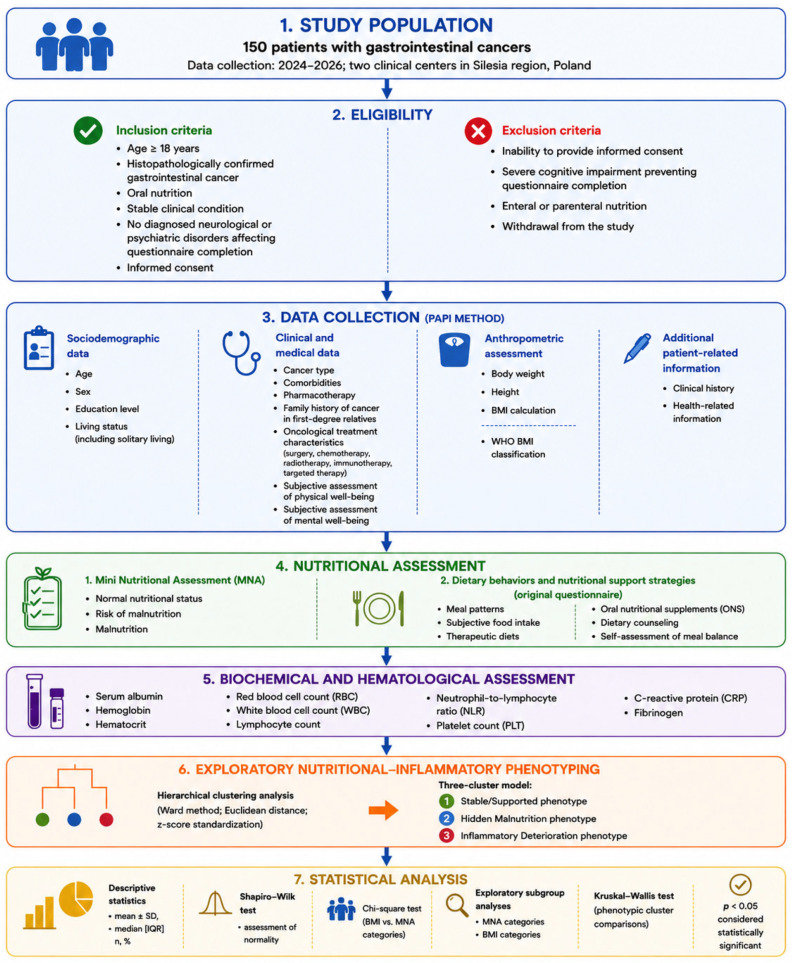
Study flowchart illustrating participant eligibility criteria, data collection procedures, nutritional assessment, biochemical and hematological evaluation, exploratory nutritional–inflammatory profiling, and statistical analyses performed in patients with gastrointestinal cancers.

The overall study design, participant eligibility criteria, data collection procedures, nutritional assessment, laboratory evaluation, and exploratory nutritional–inflammatory profiling workflow are presented in Figure 2.

### 2.2. Data Collection and Clinical Assessment

Data were collected using the Paper-and-Pencil Interview (PAPI) method, medical documentation, and laboratory records with the use of standardized and original research tools. Sociodemographic data included age, sex, education level, living status (including solitary living), and selected anthropometric parameters. Clinical and medical data included type of gastrointestinal malignancy, comorbidities, pharmacotherapy, family history of cancer among first-degree relatives, and subjective assessment of physical and mental well-being.

Body weight and height were obtained from medical records or direct measurements, and body mass index (BMI) was calculated according to the standard formula:

$$BMI=\frac{body\;weight\;(kg)}{height{\;(m)}^{2}}$$

Patients were classified according to the World Health Organization (WHO) BMI criteria.

### 2.3. Nutritional Assessment

Nutritional status was assessed using the Mini Nutritional Assessment (MNA) questionnaire. The MNA is a validated screening tool widely used in clinical and oncological practice for the identification of malnutrition and risk of malnutrition in chronically ill patients and patients with gastrointestinal malignancies. The MNA was selected because it enables multidimensional assessment of nutritional status by integrating anthropometric, dietary, functional, and self-perceived health components, facilitating early identification of patients at risk of malnutrition. The questionnaire evaluates anthropometric parameters, dietary intake, general health status, and self-perception of nutritional condition.

Based on the obtained MNA scores, patients were classified into three categories:

- normal nutritional status,
- risk of malnutrition,
- malnutrition.

Additionally, nutritional behaviors and dietary-related variables were assessed using an original questionnaire developed for the purposes of the study. Prior to the main study, the questionnaire was pilot-tested in a group of 10 patients. Based on participant feedback, several questions were modified to improve clarity and comprehensibility.

The questionnaire included selected aspects related to dietary habits, meal patterns, subjective food intake, therapeutic diets, oral nutritional supplementation, dietary counseling, and self-assessment of meal balance.

The multidimensional nutritional assessment performed in the study was intended to support the identification of nutritional disturbances and selected clinical aspects relevant to tertiary prevention in gastrointestinal cancer patients.

### 2.4. Biochemical and Hematological Assessment

Biochemical and hematological data were obtained from available medical records and laboratory test results collected during routine clinical care. Laboratory data were available only for participants with accessible biochemical and hematological records (*n* = 50). Because the availability of individual laboratory parameters varied across patients, the exact number of observations for each biochemical and hematological variable is reported in the corresponding Results subsection.

The analysis included selected biochemical and hematological parameters commonly used in the assessment of nutritional and inflammatory status in cancer patients, including serum albumin concentration, hemoglobin level, hematocrit, red and white blood cell counts, lymphocyte count, platelet count, fibrinogen concentration, neutrophil-to-lymphocyte ratio (NLR), and C-reactive protein (CRP).

The analyzed biochemical and hematological indicators were evaluated in relation to nutritional status assessed by the MNA questionnaire and were subsequently incorporated into the exploratory nutritional–inflammatory profiling analysis.

### 2.5. Statistical Analysis

Statistical analyses were performed using appropriate statistical software. Continuous variables were expressed as mean ± standard deviation (SD) or median and interquartile range (IQR), depending on variable distribution, while categorical variables were presented as numbers and percentages.

The Shapiro–Wilk test was used to assess normality of distribution. Differences between categorical variables were evaluated using the chi-square test or Fisher’s exact test, as appropriate. Associations between BMI categories and MNA nutritional status classifications were additionally evaluated using the chi-square test.

Additional exploratory subgroup analyses were performed according to:

- MNA nutritional status categories;
- BMI classification.

Moreover, an exploratory hierarchical clustering analysis was performed to identify multidimensional nutritional–inflammatory profiles among gastrointestinal cancer patients. The analysis integrated nutritional, inflammatory, biochemical, and hematological variables considered clinically relevant for nutritional and metabolic assessment in oncology patients. The following variables were included in the clustering procedure: Mini Nutritional Assessment (MNA) total score, body mass index (BMI), serum albumin concentration, C-reactive protein (CRP), fibrinogen concentration, neutrophil-to-lymphocyte ratio (NLR), hemoglobin concentration, and red blood cell (RBC) count. Although BMI constitutes one component of the MNA instrument, BMI was retained as an independent variable in the clustering procedure to explore potential discrepancies between anthropometric status and multidimensional nutritional assessment. The objective was not to validate MNA but to investigate whether preserved BMI may coexist with unfavorable nutritional and inflammatory profiles. Prior to clustering, continuous variables were standardized using z-score normalization. Hierarchical clustering analysis was performed using Ward’s linkage method with Euclidean distance measures. Based on dendrogram structure, cluster interpretability, and clinical relevance, a three-cluster model was selected for further analysis. The clustering analysis was exploratory in nature and was intended to generate hypotheses regarding potential nutritional–inflammatory profiles rather than establish validated clinical profiles.

For interpretative purposes, the identified clusters were provisionally labeled according to their dominant nutritional, inflammatory, biochemical, and hematological characteristics as follows:

- Stable/Supported profile—characterized by relatively preserved nutritional and hematological parameters and lower inflammatory activity (8%);
- Hidden Malnutrition profile—characterized by preserved or elevated BMI despite unfavorable inflammatory and biochemical markers suggestive of nutritional deterioration (46%);
- Inflammatory Deterioration profile—characterized by markedly elevated inflammatory biomarkers accompanied by severe metabolic and hematological deterioration (46%).

These labels were assigned solely to facilitate interpretation of the clustering results and should not be regarded as validated clinical profiles.

Comparisons between profile-based clusters were performed using the Kruskal–Wallis test due to non-normal distribution of inflammatory and biochemical variables. Continuous variables are presented as medians and interquartile ranges (IQRs). Statistical significance was set at *p* < 0.05.

## 3. Results

### 3.1. Characteristics of the Study Population (n = 150)

The study cohort comprised 150 patients with gastrointestinal cancers, with ages ranging from 27 to 88 years (mean age: 61.7 ± 10.6 years). The gender distribution was nearly balanced, with 76 women (50.7%) and 74 men (49.3%). Most participants had attained secondary or vocational education and were classified as overweight (48.7%) or had normal body weight (38.7%), with a smaller proportion classified as underweight (2.0%) or obese (10.7%). The predominant cancer type was colorectal cancer (63.3%), followed by gastric cancer (32.0%), with a minority diagnosed with other gastrointestinal malignancies (4.7%). Over half of the patients reported a positive family history of cancer among first-degree relatives (56.7%). Comorbidities were common, affecting 65.3% of participants, and most were on regular medication (82.0%). Subjective assessments indicated that nearly half of the patients perceived their physical (49.3%) and mental (50.7%) well-being as moderate or good (Table 1).

### 3.2. Nutritional Status and Dietary Assessment

According to the Mini Nutritional Assessment (MNA), a significant majority (79.3%) of patients were classified as at risk of malnutrition, while 2.0% were identified as malnourished. Only 18.7% demonstrated normal nutritional status. Despite these findings, 85.3% of participants subjectively considered their food intake sufficient, and most reported consuming three meals daily (81.3%). Complete meal consumption was reported by 62.7%, whereas 37.3% experienced difficulties in consuming full meals. Therapeutic diets were followed by 41.3%, and oral nutritional supplements (ONS) were used by 40.7%. Notably, only 32.0% of patients received dietary counseling during treatment. Over half of the respondents (50.7%) found it challenging to assess whether their meals were properly balanced, indicating gaps in nutritional awareness and self-evaluation (Table 2). Patients classified as being at risk of malnutrition most frequently reported moderate reductions in food intake and recent weight loss, whereas participants with normal nutritional status more commonly reported preserved food intake and no recent weight loss (Table 3).

### 3.3. Hidden Nutritional Risk Despite Preserved BMI

Despite preserved or excessive body weight, a high prevalence of nutritional risk remained evident. Among patients with normal BMI, 91.4% were classified as being at risk of malnutrition according to MNA criteria. Similarly, 79.5% of overweight patients also presented nutritional risk. Furthermore, only a minority of overweight patients presented normal nutritional status (Table 4). 

These findings suggest that BMI alone may inadequately identify clinically relevant nutritional deterioration in gastrointestinal cancer patients, as preserved body weight may mask underlying nutritional impairment.

The coexistence of nutritional risk with normal or excessive BMI may be compatible with mechanisms observed in sarcopenic obesity; however, objective body composition assessment was not performed and therefore this interpretation remains speculative.

Associations between nutritional status categories according to the MNA questionnaire and BMI classification were analysed using the chi-square test and demonstrated a statistically significant relationship (χ^2^ = 86.05; *p* < 0.001).

### 3.4. Biochemical Assessment and Nutritional-Inflammatory Profiles

The obtained laboratory findings demonstrated coexistence of inflammatory activation, nutritional deterioration, and anemia-related abnormalities. Median CRP concentration reached 67.4 mg/L, while median fibrinogen concentration was elevated to 4.4 g/L. Increased neutrophil-to-lymphocyte ratio (NLR) values (median: 4.6) additionally suggested substantial systemic inflammatory response. Simultaneously, reduced median albumin concentration (29.1 g/L), hemoglobin level (11.1 g/dL), and RBC count (3.7 × 10^6^/µL) indicated coexistence of nutritional deterioration and anemia-related disturbances in the analyzed patients (Table 5). Collectively, the biochemical findings support the coexistence of nutritional risk and systemic inflammatory activation in gastrointestinal cancer patients.

Comparative analysis between the identified nutritional–inflammatory profiles demonstrated significant heterogeneity of inflammatory, biochemical, and hematological parameters among gastrointestinal cancer patients.

**Figure 3:**
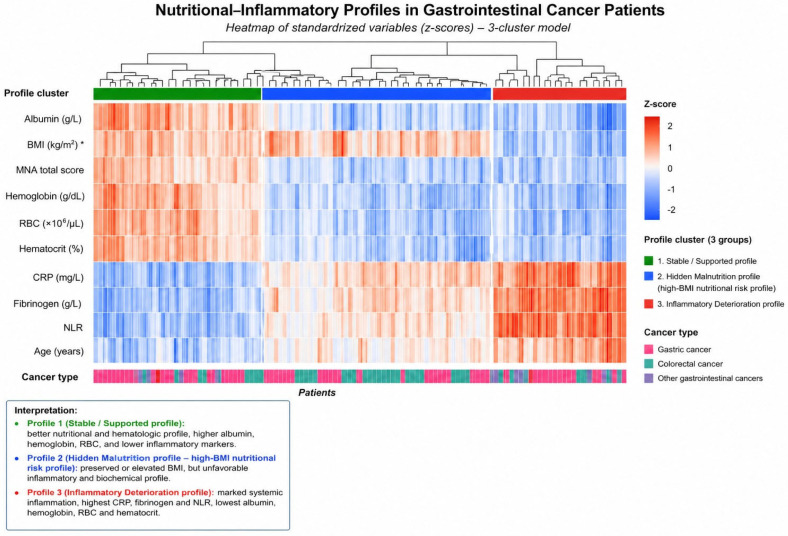
Exploratory nutritional–inflammatory profiles identified in patients with gastrointestinal cancers using hierarchical clustering analysis based on MNA score, BMI, inflammatory biomarkers, and selected biochemical and hematological indicators. Rows represent standardized values (z-scores) of the analyzed variables. Higher values (red) indicate higher levels of the respective variable, whereas lower values (blue) indicate lower levels. * BMI is presented as a continuous standardized variable (z-score) and was included to explore potential discrepancies between anthropometric status and multidimensional nutritional assessment. WHO BMI categories are reported separately in Table 1 and were not included in the heatmap annotations to avoid duplication. The identified profiles should be interpreted as exploratory and hypothesis-generating. BMI is presented as a standardized continuous variable (z-score) used in the clustering analysis, whereas WHO BMI categories are reported separately in Table 1.

Figure 3 illustrates three exploratory nutritional–inflammatory profiles identified through hierarchical clustering based on nutritional, inflammatory, biochemical, and hematological variables.

The first subgroup, referred to as the Stable/Supported profile, was characterized by relatively preserved nutritional and hematological parameters, higher albumin concentration, and lower inflammatory activity. This subgroup demonstrated the most favorable nutritional and metabolic profile within the analyzed cohort and may correspond to patients with relatively preserved nutritional and inflammatory status. However, these observations should be interpreted as exploratory and hypothesis-generating and do not imply clinically validated patient stratification.

The second identified profile, termed the hidden malnutrition profile, was characterized by preserved or excessive BMI despite low or intermediate MNA scores accompanied by elevated inflammatory markers, particularly CRP and NLR, together with reduced albumin concentration. This profile may indicate the presence of occult nutritional deterioration despite preserved body weight; however, objective body composition assessment was not performed and therefore this interpretation remains speculative.

Finally, the Inflammatory Deterioration profile included patients presenting the most pronounced metabolic and inflammatory disturbances, including markedly elevated CRP and fibrinogen concentrations, high NLR values, and anemia-related abnormalities. This profile was additionally characterized by the lowest albumin, hemoglobin, and RBC values among the identified clusters. Such a pattern may be compatible with inflammation-driven metabolic deterioration and mechanisms associated with cancer-related cachexia; however, direct assessment of body composition was not available.

The identified profiles should be considered exploratory and hypothesis-generating. Although hierarchical clustering revealed distinct nutritional–inflammatory patterns, further validation in larger and independent cohorts is required before these profiles can be considered clinically applicable.

Significant differences between clusters were observed for BMI (*p* < 0.001), CRP (*p* < 0.001), fibrinogen (*p* = 0.005), albumin (*p* < 0.001), hemoglobin (*p* < 0.001), and RBC count (*p* < 0.001). In contrast, MNA score (*p* = 0.267) and NLR (*p* = 0.149) did not significantly differ between clusters. The inflammatory deterioration profile demonstrated the highest CRP and fibrinogen concentrations together with the lowest albumin, hemoglobin, and RBC values, indicating pronounced systemic inflammatory activation accompanied by severe metabolic and hematological deterioration. Patients classified within the hidden malnutrition profile demonstrated preserved or elevated BMI despite unfavorable inflammatory and biochemical profiles, supporting the presence of occult nutritional deterioration masked by preserved body weight. In contrast, the stable/supported profile demonstrated the most favorable inflammatory and nutritional profile.

## 4. Discussion

### 4.1. Nutritional Risk and Malnutrition in Patients with Gastrointestinal Cancers

The present study demonstrated a high prevalence of impaired nutritional status among patients with gastrointestinal cancers. According to the MNA classification, more than 80% of participants were either at risk of malnutrition or already malnourished. These findings confirm that nutritional deterioration remains one of the major clinical challenges in gastrointestinal oncology and emphasize the importance of routine nutritional screening as part of tertiary prevention and supportive cancer care [23,24,25].

The present study intentionally analyzed patients with different gastrointestinal malignancies to identify nutritional and inflammatory patterns common across gastrointestinal cancers rather than tumor-specific characteristics. Nevertheless, colorectal, gastric, pancreatic, liver, and esophageal cancers differ substantially with respect to disease biology, treatment strategies, anatomical consequences, and nutritional impairment. Therefore, the present findings should be interpreted as reflecting shared features of gastrointestinal oncology rather than disease-specific nutritional characteristics. Future studies involving larger and more homogeneous tumor-specific cohorts are warranted.

Cancer-related malnutrition is a multifactorial syndrome associated with reduced dietary intake, metabolic dysregulation, chronic systemic inflammation, treatment-related toxicity, and progressive catabolism. Anticancer treatment modalities, including surgery, chemotherapy, radiotherapy, and systemic therapies, may additionally contribute to nutritional deterioration through gastrointestinal symptoms, appetite loss, altered metabolism, and systemic inflammatory activation. Patients with gastrointestinal malignancies appear particularly vulnerable due to impaired digestion and absorption, anorexia, nausea, dysphagia, altered taste perception, and cancer cachexia. Similar observations were reported in previous oncology studies, where malnutrition and nutritional risk were associated with reduced treatment tolerance, prolonged hospitalization, poorer quality of life, and increased mortality in gastrointestinal cancer patients [24,25,26,27,28]. Previous reports indicated that the prevalence of nutritional risk in gastrointestinal cancers may range from approximately 40% to over 70%, depending on tumor location, disease stage, and assessment method used [23,26,29].

The present findings are also consistent with previous studies conducted among colorectal cancer patients, where nutritional disturbances and unfavorable lifestyle-related behaviors frequently coexisted during oncological treatment and survivorship. Our previous observations similarly demonstrated that tertiary prevention behaviors among colorectal cancer patients remain heterogeneous and frequently insufficiently supported by professional nutritional guidance [30]. Furthermore, previous studies published in *Nutrients* and *Cancers* emphasized that nutritional status, lifestyle-related factors, and supportive care strategies may substantially influence quality of life and treatment-related functioning among oncology patients [27,28,30]. These findings collectively support the growing recognition that nutritional care should not be limited exclusively to advanced cachexia but integrated into comprehensive oncology management from earlier stages of treatment.

### 4.2. Limitations of BMI and the Importance of Multidimensional Nutritional Assessment

An important observation of the present study was the coexistence of high nutritional risk with predominantly normal or excessive body weight. Despite 48.7% of participants being classified as overweight and 38.7% presenting normal body weight, 79.3% remained at risk of malnutrition according to MNA assessment. These findings support previous reports indicating that BMI alone may inadequately reflect nutritional deterioration in oncology patients.

Recent studies increasingly emphasize the phenomenon of sarcopenic obesity, characterized by the coexistence of excessive adiposity and skeletal muscle depletion. Sarcopenic obesity has been associated with poorer prognosis, increased postoperative complications, reduced survival, systemic inflammation, and treatment toxicity in cancer patients.

Previous studies have shown that sarcopenic obesity represents a clinically distinct condition associated with systemic inflammation, oxidative stress, insulin resistance, impaired functional status, and poorer oncological outcomes across multiple cancer types, including gastrointestinal malignancies [31,32,33]. Similarly, increasing evidence suggests that visceral adiposity, myosteatosis, and ectopic fat accumulation may adversely affect prognosis in gastric and other gastrointestinal cancers, emphasizing that preserved or elevated BMI may mask substantial metabolic and nutritional disturbances [33,34].

The growing interest in body composition assessment additionally reflects limitations of conventional anthropometric evaluation. Previous studies evaluating body composition have demonstrated that excessive body weight may coexist with skeletal muscle depletion and adverse metabolic alterations, limiting the usefulness of BMI as a standalone indicator of nutritional status [34,35]. These findings may partially explain why many patients in the present study remained at nutritional risk despite normal or excessive body weight.

The present findings therefore support the clinical usefulness of multidimensional nutritional assessment tools such as the MNA questionnaire, which may identify nutritional impairment even in patients without low body weight. From a clinical perspective, reliance solely on BMI may lead to underrecognition of nutritional deterioration and delayed nutritional intervention in gastrointestinal oncology patients. These observations further support current recommendations advocating the integration of nutritional screening with clinical, anthropometric, and functional assessment in oncology practice [35].

Nevertheless, body composition was not assessed in the present study and therefore no direct conclusions regarding sarcopenia or sarcopenic obesity can be drawn.

### 4.3. Dietary Behaviors, Nutritional Strategies, and Patient Awareness

The dietary assessment performed in the present study revealed several clinically important gaps in nutritional management among gastrointestinal cancer patients. Although 85.3% of participants subjectively perceived their food intake as sufficient, objective assessment using the MNA questionnaire simultaneously indicated a high prevalence of nutritional risk. This discrepancy may suggest limited nutritional awareness among oncology patients and difficulties in recognizing early nutritional deterioration.

Previous studies similarly reported that self-perceived dietary adequacy frequently differs from objective nutritional assessment, particularly in patients with chronic diseases and cancer [36,37]. Previous studies conducted among colorectal cancer survivors demonstrated that dietary modifications are frequently introduced independently following diagnosis, often without professional nutritional supervision [36]. Many patients seek nutritional information from non-medical sources, contributing to uncertainty regarding meal balance, nutritional adequacy, and evidence-based dietary recommendations [38]. These findings correspond with the present results, where 50.7% of participants reported difficulties assessing whether their meals were appropriately balanced.

The present study additionally demonstrated relatively limited implementation of nutritional strategies during oncological treatment. Only 40.7% of participants reported using oral nutritional supplements, whereas therapeutic diets were followed by 41.3% of patients. Furthermore, complete meal consumption was declared by 62.7% of participants, while 37.3% reported difficulties consuming full meals. Dietary counseling during treatment was reported by only 32.0% of respondents. Similar deficiencies in nutritional support and patient education have been described in previous studies evaluating nutritional care among oncology patients and cancer survivors [38,39,40,41].

Importantly, nutritional deterioration may negatively affect not only treatment outcomes but also physical functioning, fatigue severity, independence, and overall quality of life in cancer patients. Current evidence increasingly indicates that individualized dietary counseling and nutritional support may improve nutritional intake, treatment tolerance, maintenance of functional status, and patient quality of life [40,41,42,43]. Nutritional interventions are also recognized as important supportive measures aimed at reducing treatment-related complications and preserving patient autonomy throughout anticancer therapy [41,42,43]. In this context, the present findings suggest that nutritional support remains insufficiently integrated into routine oncological practice despite its recognized clinical importance.

### 4.4. Nutritional Counseling and Multidisciplinary Supportive Care

The low percentage of patients receiving professional dietary counseling observed in the present study highlights a potentially important gap in supportive oncology care. Despite the high prevalence of nutritional risk, only 32% of participants reported receiving dietary consultation during treatment. These findings appear clinically relevant considering current recommendations emphasizing early nutritional intervention and multidisciplinary supportive care in oncology patients [25,43].

Recent supportive care models increasingly underline the importance of integrating oncologists, dietitians, psycho-oncologists, rehabilitation specialists, and palliative care teams into comprehensive cancer management [27,28]. Scotté et al. emphasized that supportive care should constitute an integral component of anticancer treatment rather than an optional addition introduced exclusively in advanced disease stages [27]. Similarly, Gouldthorpe et al. highlighted the importance of supportive and palliative interventions throughout the entire cancer continuum, including nutritional management and symptom control [28].

Nutritional counseling may additionally represent an important “teachable moment” during cancer treatment, when patients become more motivated to modify dietary and lifestyle-related behaviors [36]. However, without appropriate professional guidance, dietary modifications may become restrictive, unbalanced, or inconsistent with evidence-based recommendations. The present findings suggest that many patients remain uncertain regarding nutritional adequacy and meal balance, further emphasizing the need for structured nutritional education integrated into routine oncological care [36,39].

Previous integrative reviews also demonstrated that nutritional support in oncology and palliative care should extend beyond simple caloric supplementation and include counseling, symptom-oriented dietary strategies, psychosocial support, and individualized patient-centered care. Collectively, these observations support the concept that nutritional care should be considered an essential component of tertiary prevention and quality-of-life-oriented oncology management [38,42,44].

### 4.5. Inflammatory and Hematological Biomarkers in the Context of Nutritional Status

The biochemical findings obtained in the subgroup with available laboratory data additionally support the coexistence of nutritional deterioration and systemic inflammatory activation in gastrointestinal cancer patients. Mean serum albumin concentration was reduced to 29.0 ± 6.7 g/L, while mean CRP concentration reached 79.1 ± 76.0 mg/L. Furthermore, elevated fibrinogen levels (4.5 ± 1.5 g/L) and increased NLR values (5.7 ± 5.3) suggested substantial systemic inflammatory activation in the analyzed subgroup.

Reduced hemoglobin concentration (11.0 ± 1.9 g/dL), hematocrit (33.6 ± 5.6%), and red blood cell count (3.7 ± 0.6 × 10^6^/µL) additionally indicated the coexistence of anemia-related abnormalities frequently accompanying cancer progression and chronic inflammation. These findings are biologically consistent with mechanisms underlying cancer-related malnutrition and cachexia.

Chronic inflammation plays a central role in cancer-associated metabolic disturbances through activation of catabolic pathways, appetite suppression, altered protein metabolism, and skeletal muscle wasting [45]. Elevated inflammatory markers such as CRP and fibrinogen have previously been associated with poorer prognosis, cachexia progression, lower treatment tolerance, and reduced survival in gastrointestinal malignancies [46]. Similarly, reduced serum albumin concentration may reflect both nutritional deterioration and systemic inflammatory burden, as albumin synthesis is strongly affected by inflammatory cytokine activity [47].

Particularly noteworthy in the present study was the inclusion of the neutrophil-to-lymphocyte ratio (NLR), which is increasingly recognized as an accessible biomarker of systemic inflammatory response in oncology. Elevated NLR values observed in the analyzed subgroup may indicate immune dysregulation accompanying cancer progression. Previous studies demonstrated associations between inflammatory and anemia-related indices and colorectal cancer burden, highlighting the clinical significance of integrated inflammatory assessment in oncology patients [48]. Similarly, combined inflammatory markers integrating CRP and NLR have been reported to provide prognostic value in gastric cancer patients [49].

Recent meta-analyses additionally demonstrated that elevated NLR and PLR values are associated with poorer overall survival and progression-free survival in gastric cancer patients, further supporting the prognostic role of systemic inflammatory markers in gastrointestinal oncology [50]. Furthermore, previous studies evaluating colorectal cancer patients identified elevated NLR and reduced albumin concentration as independent predictors of postoperative complications and poorer clinical outcomes [51].

These findings collectively suggest that nutritional deterioration and systemic inflammation should be evaluated simultaneously in gastrointestinal oncology patients, as both processes appear closely interconnected and clinically relevant. Integration of nutritional assessment with selected inflammatory and hematological biomarkers may therefore improve identification of patients requiring intensified supportive and nutritional interventions.

### 4.6. Nutritional–Inflammatory Profiles and Metabolic Heterogeneity

An important aspect of the present study was the identification of exploratory nutritional–inflammatory profiles based on multidimensional clustering analysis integrating nutritional, inflammatory, biochemical, and hematological parameters. Hierarchical clustering revealed substantial metabolic heterogeneity among gastrointestinal cancer patients despite apparent similarities in body weight or general clinical status.

Particularly noteworthy was the identification of the hidden malnutrition profile, characterized by preserved or elevated BMI despite unfavorable inflammatory and biochemical nutritional profiles, including elevated CRP and fibrinogen concentrations together with reduced albumin and hemoglobin levels. These findings support growing evidence suggesting that excessive or normal body weight may mask significant metabolic and nutritional deterioration in oncology patients [52,53,54]. However, because objective body composition assessment was not performed, this profile should not be interpreted as direct evidence of sarcopenic obesity. Rather, it may be compatible with mechanisms observed in sarcopenic obesity; however, this interpretation remains speculative.

The identified inflammatory deterioration profile demonstrated markedly elevated inflammatory biomarkers accompanied by profound hematological and biochemical abnormalities, reflecting mechanisms consistent with cancer-related cachexia and systemic metabolic dysregulation. Chronic inflammatory activation is increasingly recognized as a central driver of muscle wasting, altered protein turnover, appetite suppression, and progressive functional decline in cancer patients [55,56]. Previous studies evaluating inflammatory and cachexia-related biomarkers similarly demonstrated associations between elevated CRP, NLR, hypoalbuminemia, anemia, and unfavorable oncological outcomes [42,53,55].

Importantly, MNA score alone did not significantly differentiate the identified profile-based clusters, whereas biochemical and inflammatory markers demonstrated substantial intergroup variability. Interestingly, although MNA identified a generally high prevalence of nutritional risk, the clustering procedure revealed additional metabolic heterogeneity driven mainly by inflammatory and biochemical markers rather than by MNA score alone. These observations may suggest that isolated nutritional screening tools may insufficiently capture complex metabolic deterioration accompanying gastrointestinal malignancies. Simultaneous integration of nutritional screening with inflammatory and hematological biomarkers may therefore provide a more comprehensive assessment of nutritional risk and supportive care needs in oncology patients [22,52,53].

The stable/supported profile identified in the present study demonstrated more favorable inflammatory and biochemical profiles together with relatively preserved hematological status. This profile may reflect patients with relatively preserved nutritional and metabolic status; however, the factors underlying this pattern cannot be determined within the framework of the present cross-sectional study.

The concept of nutritional–inflammatory profiling corresponds with the growing interest in precision supportive oncology and individualized metabolic assessment in cancer patients. Recent oncology research increasingly emphasizes that nutritional deterioration should not be interpreted exclusively through anthropometric parameters but rather as a multidimensional metabolic process involving systemic inflammation, altered body composition, immune dysregulation, and functional decline [17,54,55,56].

Collectively, the present findings suggest that multidimensional profile-based assessment may improve identification of gastrointestinal cancer patients requiring intensified nutritional monitoring and individualized supportive interventions beyond standard BMI-based evaluation alone.

The identified profiles should be considered exploratory and hypothesis-generating. Because the clustering procedure was performed using an unsupervised analytical approach and objective body composition assessment was not available, these findings require validation in larger, independent, and preferably prospective cohorts before their potential use in clinical decision-making.

### 4.7. Clinical Implications and Future Directions

The present findings highlight the importance of multidimensional nutritional assessment in gastrointestinal oncology. The coexistence of nutritional risk, inflammatory activation, anemia-related abnormalities, and insufficient nutritional support suggests that nutritional care remains underrecognized in routine oncology practice.

Routine screening using validated tools such as the MNA questionnaire, combined with dietary evaluation and selected biochemical markers, may facilitate earlier identification of patients at increased nutritional and metabolic risk. Early nutritional intervention, individualized dietary counseling, oral nutritional supplementation, and systematic monitoring of inflammatory biomarkers may contribute to improved treatment tolerance, maintenance of functional status, reduction in complications, and better quality of life during anticancer therapy.

The exploratory identification of nutritional–inflammatory profiles further suggests that gastrointestinal cancer patients represent a metabolically heterogeneous population. Recognition of exploratory profiles such as Hidden Malnutrition or Inflammatory Deterioration may potentially facilitate earlier identification of patients requiring intensified nutritional monitoring, supportive care, and individualized interventions beyond standard BMI-based assessment.

Future studies should include more detailed analyses of relationships between nutritional status, dietary behaviors, inflammatory biomarkers, and selected clinical and sociodemographic factors in patients with gastrointestinal cancers. Particular attention should be paid to variables such as sex, age, educational level, place of residence, comorbidities, cancer-related characteristics, and treatment-related factors.

Further research is also warranted to evaluate quality of life, symptom burden, and both pharmacological and non-pharmacological treatment adherence in relation to nutritional status and supportive care needs. Such multidimensional analyses may contribute to better identification of patients requiring individualized nutritional and supportive interventions during anticancer treatment.

Additionally, prospective and longitudinal studies assessing changes in nutritional status, body composition, inflammatory activation, and clinical outcomes over time are needed. Further investigation of sarcopenic obesity, metabolic dysregulation, and personalized nutritional interventions may improve understanding of nutritional deterioration mechanisms in gastrointestinal cancers and support the development of more individualized tertiary prevention strategies.

## 5. Strengths and Limitations

### 5.1. Strengths

The present study has several strengths. First, a multidimensional approach was applied, integrating nutritional screening, dietary assessment, clinical characteristics, and biochemical indicators in patients with gastrointestinal cancers. Such a comprehensive evaluation allowed assessment not only of nutritional status itself, but also of selected dietary behaviors, supportive nutritional strategies, and inflammation-related biomarkers.

The study combined subjective patient-reported nutritional information with objective laboratory and hematological parameters, including CRP, fibrinogen, albumin concentration, and NLR. This broader clinical perspective increases the practical and translational relevance of the findings in the context of supportive oncology and tertiary prevention.

The study addressed clinically important yet still underrecognized aspects of oncological care, including nutritional counseling, patient awareness, and implementation of nutritional support during treatment. The inclusion of tertiary prevention concepts and multidisciplinary supportive care additionally strengthens the practical implications of the presented findings.

Another strength of the study is the inclusion of both nutritional and inflammatory parameters, which allowed simultaneous evaluation of metabolic disturbances and systemic inflammatory activation in gastrointestinal cancer patients. Furthermore, the use of the validated MNA questionnaire increased the reliability and clinical applicability of nutritional risk assessment.

An additional strength of the study is the exploratory identification of nutritional–inflammatory profiles using hierarchical clustering analysis. This multidimensional approach enabled the integration of nutritional, inflammatory, biochemical, and hematological variables, revealing distinct patterns of nutritional and metabolic heterogeneity that may not be fully captured by conventional nutritional screening tools alone.

The identification of Stable/Supported, Hidden Malnutrition, and Inflammatory Deterioration profiles further highlights the heterogeneity of nutritional and metabolic disturbances among patients with gastrointestinal cancers and may contribute to the development of more individualized supportive care strategies in future research.

### 5.2. Limitations

Several limitations should be acknowledged. First, the cross-sectional design precludes causal inference between nutritional status, dietary behaviors, inflammatory activation, and biochemical abnormalities. Moreover, the identified nutritional–inflammatory profiles were derived using exploratory hierarchical clustering and should be regarded as hypothesis-generating rather than definitive clinical classifications. Because BMI constitutes one component of the MNA score, some overlap between variables included in the clustering procedure cannot be excluded, although BMI was intentionally retained to explore discrepancies between anthropometric status and multidimensional nutritional assessment. Consequently, validation in larger, independent, and preferably prospective cohorts is required before broader clinical application.

Second, biochemical analyses were available only for a subgroup of participants, and the study population comprised patients with different gastrointestinal malignancies. Therefore, the laboratory-related findings should be interpreted with appropriate caution. Owing to the limited sample size within individual cancer subgroups and incomplete information regarding TNM stage, surgical treatment, recurrence status, and treatment phase, subgroup analyses were not feasible and the influence of disease- and treatment-related factors on nutritional status could not be fully assessed.

Third, the dietary questionnaire was developed specifically for this study and underwent pilot testing only. Full psychometric validation was not performed, and therefore findings derived from this instrument should be interpreted with caution. Likewise, objective body composition assessment was unavailable, precluding formal diagnosis of sarcopenia or sarcopenic obesity. Consequently, interpretations regarding mechanisms compatible with sarcopenic obesity remain speculative. In addition, the study lacked longitudinal follow-up, preventing evaluation of temporal changes in nutritional status and supportive care needs.

Finally, several nutritional and dietary variables were based on self-reported patient responses and may therefore be affected by recall bias. Further prospective studies involving larger and more homogeneous patient populations are needed to validate the identified nutritional–inflammatory profiles and better characterize the relationships between nutritional deterioration, systemic inflammation, and supportive care needs in gastrointestinal oncology.

## 6. Conclusions

The present study demonstrated a high prevalence of nutritional risk among patients with gastrointestinal cancers, despite predominantly normal or excessive body weight observed in the study population. The findings additionally revealed important gaps in nutritional care, including limited dietary counseling and insufficient implementation of nutritional support strategies during anticancer treatment.

The coexistence of nutritional risk, inflammatory activation, and anemia-related abnormalities highlights the need for routine multidimensional nutritional assessment integrating validated screening tools, dietary evaluation, and selected biochemical markers in gastrointestinal oncology patients.

The exploratory nutritional–inflammatory profiling approach additionally identified distinct nutritional and inflammatory patterns, including Hidden Malnutrition and Inflammatory Deterioration profiles, emphasizing the heterogeneity of nutritional and inflammatory disturbances among gastrointestinal cancer patients.

A multidimensional assessment integrating nutritional screening, dietary evaluation, inflammatory biomarkers, and exploratory profile-based clustering may facilitate future research on individualized nutritional assessment and supportive care. However, the identified nutritional-inflammatory profiles should currently be regarded as exploratory and hypothesis-generating and require validation in larger prospective studies before clinical implementation.

Further prospective studies are required to evaluate the long-term clinical impact of integrated nutritional and supportive care strategies in patients with gastrointestinal cancers.

## Figures and Tables

**Table 1:** Characteristics of the Study Population (*n* = 150).

Variable	Category	*n* (%)
Sex	Female	76 (50.7)
	Male	74 (49.3)
Education level	Primary education	2 (1.3)
	Vocational education	47 (31.3)
	Secondary education	75 (50.0)
	Higher education	26 (17.3)
BMI classification	Underweight	3 (2.0)
	Normal body weight	58 (38.7)
	Overweight	73 (48.7)
	Obesity	16 (10.7)
Living alone	Yes	74 (49.3)
	No	76 (50.7)
Primary cancer site	Colorectal cancer	95 (63.3)
	Gastric cancer	48 (32.0)
	Other gastrointestinal cancers	7 (4.7)
Oncological treatment	Surgery	139 (92.7)
	Chemotherapy	92 (61.3)
	Radiotherapy	62 (41.3)
	Immunotherapy	22 (14.7)
	Targeted therapy	17 (11.3)
Family history of cancer in first-degree relatives	Yes	85 (56.7)
	No	65 (45.3)
Comorbidities	Yes	98 (65.3)
	No	52 (34.7)
Medication use	Yes	123 (82.0)
	No	27 (18.0)
Subjective physical well-being	Poor	8 (5.3)
	Moderate	63 (42.0)
	Good	74 (49.3)
	Very good	5 (3.3)
Subjective mental well-being	Poor	7 (4.7)
	Moderate	54 (36.0)
	Good	76 (50.7)
	Very good	13 (8.7)

**Table 2:** Nutritional Status and Dietary Assessment of the Study Population According to the Mini Nutritional Assessment (MNA) and Selected Dietary Behaviors.

Variable	Category	*n* (%)
Nutritional status according to MNA	Normal nutritional status	28 (18.7)
	Risk of malnutrition	119 (79.3)
	Malnutrition	3 (2.0)
Subjective assessment of food intake		
	Too high	5 (3.3)
	Sufficient	128 (85.3)
	Too low	17 (11.3)
Number of meals per day		
	
	
	<3	3 (2.0)
	3	122 (81.3)
	4	20 (13.3)
	5	5 (3.3)
	>5	0 (0.0)
Complete meal consumption	Yes	94 (62.7)
	No	56 (37.3)
Therapeutic diet use	Yes	62 (41.3)
	No	88 (58.9)
Oral nutritional supplements (ONS) use	Yes	61 (40.7)
	No	89 (59.3)
Dietary counseling during treatment	Yes	48 (32.0)
	No	102 (68.0)
Self-assessment of meal balance	Yes	57 (38.0)
	No	17 (11.3)
	Difficult to assess/uncertain	76 (50.7)

**Table 3:** Distribution of responses to selected Mini Nutritional Assessment (MNA) items according to nutritional status categories.

MNA Item	Normal Nutritional Status *n* = 28	Risk of Malnutrition *n* = 119	Malnutrition *n* = 3
Food intake decline			
	Moderate (*n* = 3; 10.7%)	Moderate (*n* = 75; 63%)	Moderate (*n* = 0; 0%)
	None (*n* = 25; 89.3%)	None (*n* = 44; 37%)	None (*n* = 3; 100%)
Weight loss	>3 kg (*n* = 0; 0%)	>3 kg (*n* = 42; 35.3%)	>3 kg (*n* = 2; 66.7%)
	1–3 kg (*n* = 12; 42.9%) Unknown (*n* = 2; 7.1%) None (*n* = 14; 50%)	1–3 kg (*n* = 27; 22.7%) Unknown (*n* = 44; 37%) None (*n* = 6; 5%)	1–3 kg (*n* = 0; 0%) Unknown (*n* = 0; 0%) None (*n* = 1; 33.3%)
Use of more than 3 medications	Yes (*n* = 7; 25%)	Yes (*n* = 65; 54.6%)	Yes (*n* = 3; 100%)
	No (*n* = 21; 75%)	No (*n* = 54; (45.4%)	No (*n* = 0; 0%)

**Table 4:** Association Between BMI Classification and Nutritional Status According to the Mini Nutritional Assessment (MNA).

BMI Category	Normal Nutritional Status *n* (%)	Risk of Malnutrition *n* (%)	Malnutrition *n* (%)
Underweight (*n* = 3)	0 (0.0)	1 (33.3)	2 (66.7)
Normal body weight (*n* = 58)	4 (6.9)	53 (91.4)	1 (1.7)
Overweight (*n* = 73)	15 (20.5)	58 (79.5)	0 (0.0)
Obesity (*n* = 16)	9 (56.2)	7 (43.8)	0 (0.0)

**Chi-square test: χ^2^ = 86.05; *p* < 0.001.**

**Table 5:** Comparison of biochemical and nutritional parameters between profile-based clusters (Kruskal–Wallis test).

Variable	Total Cohort	Stable/Supported Profile	Hidden Malnutrition Profile	Inflammatory Deterioration Profile	*p*-Value
MNA total score (*n* = 150)	21.5 (19.0–23.0)	21.5 (19.8–22.0)	21.5 (20.8–23.8)	21.8 (20.5–22.1)	0.267
BMI (kg/m^2^) (*n* = 150)	25.4 (22.9–28.0)	24.1 (22.1–25.8)	27.7 (25.9–29.3)	24.3 (21.9–26.1)	<0.001
CRP (mg/L) (*n* = 50)	67.4 (31.2–112.8)	35.0 (18.7–59.5)	74.8 (38.6–102.7)	239.8 (222.7–272.4)	<0.001
NLR (*n* = 50)	4.6 (2.7–6.8)	3.0 (2.2–6.2)	4.5 (2.7–6.7)	8.0 (5.9–11.0)	0.149
Fibrinogen (g/L) (*n* = 50)	4.4 (3.4–5.3)	3.8 (3.3–4.3)	4.6 (3.5–5.7)	6.7 (6.3–7.0)	0.005
Albumin (g/L) (*n* = 50)	29.1 (25.0–33.0)	32.2 (30.2–34.4)	27.5 (24.3–30.9)	18.0 (16.1–19.3)	<0.001
Hemoglobin (g/dL) (*n* = 50)	11.1 (9.9–12.5)	12.3 (11.8–13.4)	9.9 (9.3–11.0)	8.1 (8.0–8.2)	<0.001
RBC (×10^6^/µL) (*n* = 50)	3.7 (3.3–4.2)	4.2 (4.0–4.4)	3.4 (3.2–3.6)	2.7 (2.6–2.9)	<0.001

## Data Availability

The data presented in this study are available within the article. Additional data are available from the corresponding author upon request.
